# The effect of stereotactic body radiotherapy (SBRT) using flattening filter‐free beams on cardiac implantable electronic devices (CIEDs) in clinical situations

**DOI:** 10.1002/acm2.12873

**Published:** 2020-04-11

**Authors:** Hossein Aslian, Tomas Kron, Troy Watts, Cagla Akalanli, Nicholas Hardcastle, Peta Lonski, Atousa Montaseri, Barry Hay, James Korte, Kemal Berk, Francesco Longo, Mara Severgnini

**Affiliations:** ^1^ Department of Physics University of Trieste Trieste Italy; ^2^ Physical Sciences Peter MacCallum Cancer Centre Melbourne Australia; ^3^ Department of Cardiology Royal Melbourne Hospital Melbourne Australia; ^4^ Italian National Institute of Nuclear Physics (INFN) sezione di Trieste Trieste Italy; ^5^ Department of Medical Physics Azienda Sanitaria Universitaria Integrata di Trieste Trieste Italy

**Keywords:** cardiac implantable electronic device (CIED), flattening filter‐free (FFF) beams, stereotactic body radiotherapy (SBRT), volumetric‐modulated arc therapy (VMAT)

## Abstract

**Purpose:**

This study focused on determining risks from stereotactic radiotherapy using flattening filter‐free (FFF) beams for patients with cardiac implantable electronic device (CIEDs). Two strategies were employed: a) a retrospective analysis of patients with CIEDs who underwent stereotactic radiosurgery (SRS)/SBRT at the Peter MacCallum Cancer Centre between 2014 and 2018 and b) an experimental study on the impact of FFF beams on CIEDs.

**Methods:**

A retrospective review was performed. Subsequently, a phantom study was performed using 30 fully functional explanted CIEDs from two different manufacturers. Irradiation was carried out in a slab phantom with 6‐MV and 10‐MV FFF beams. First, a repetition‐rate test (RRT) with a range of beam pulse frequencies was conducted. Then, multifraction SBRT (48 Gy/4 Fx) and single‐fraction SBRT (28 Gy/1 Fx) treatment plans were used for lung tumors delivered to the phantom.

**Results:**

Between 2014 and 2018, 13 cases were treated with an FFF beam (6 MV, 1400 MU/min or 10 MV, 2400 MU/min), and 15 cases were treated with a flattening filter (FF) beam (6 MV, 600 MU/min). All the devices were positioned outside the treatment field at a distance of more than 5 cm, except for one case, and no failures were reported due to SBRT/SRS. In the phantom rep‐rate tests, inappropriate sensing occurred, starting at a rep‐rate of 1200 MU/min. Cardiac implantable electronic device anomalies during and after delivering VMAT‐SBRT with a 10‐MV FFF beam were observed.

**Conclusions:**

The study showed that caution should be paid to managing CIED patients when they undergo SBRT using FFF beams, as it is recommended by AAPM TG‐203. Correspondingly, it was found that for FFF beams although there is small risk from dose‐rate effects, delivering high dose of radiation with beam energy greater than 6 MV and high‐dose rate to CIEDs positioned in close vicinity of the PTV may present issues.

## 1 Introduction

Stereotactic radiosurgery (SRS) was first conceptualized by Lars Leksell^1^, ^2^ in 1950 as a single‐fraction ablative radiotherapy for intracranial tumors. Years later, in 1991, an extension of this concept was applied to extracranial tumors by Blomgren and Lax,^1^, ^2^ called stereotactic body radiation therapy (SBRT), which has recently been renamed stereotactic ablative body radiotherapy (SABR). Today, SBRT has become an effective, widespread modality for the treatment of both primary and metastatic cancers.^3^, ^4^

Over the last few decades, there has been an ever‐increasing number of patients undergoing cardiac implantable electronic device (CIED) implantation to improve quality of life and prolong survival among those suffering from cardiovascular disease.^5^, ^6^ As the average patient age increases, the number of cancer patients and comorbid cardiovascular disease also increases.^7^

For instance, the total number of patients treated across PeterMac's five Victorian campuses has been reasonably steady at 6500 per year. However, our retrospective analysis confirmed a significant continuous increase in the fraction of patients presenting with implantable devices (from 35 CIED patients in 2014 to 143 CIED patients in 2018). This yields an increase from 0.5 to 2% of patients needing consideration for CIEDs.

Due to this fact, many studies have focused on the effect of radiotherapy on patients with CIEDs, and many aspects of this field have been investigated in the literature.^8^, ^9^, ^10^ However, guidelines^11^, ^12^ and reviews^9^, ^13^ mainly address the management of patients with CIEDs undergoing conventional radiotherapy.^14^, ^15^ The first recommendation from the AAPM on the management of radiotherapy patients with CIEDs dates back to 1994 (TG‐34).^16^ During the revision of this paper, a new AAPM practice guideline to manage CIED patients receiving radiotherapy (TG‐203) has been published.^17^ In this report, several related challenges to this topic were firstly discussed in detail and general and specific recommendations were then made to manage patients treated with conventional and modern radiotherapy techniques and technologies.

A recent review^14^ discussed in detail some of the different characteristics of SBRT/SRS compared to conventional radiotherapy that might indicate that special considerations are required for the safety of patients with CIEDs. These features are a) higher dose per fraction in SBRT, which might result in a higher dose per fraction to the CIED, b) SBRT‐dedicated treatment technologies (e.g., CyberKnife, Gamma‐Knife, and VERO), c) different techniques to achieve conformal doses (such as multiple static fields/arcs and noncoplanar geometries), d) different out‐of‐field doses,^18^ higher monitor units (MUs) in modulated techniques (e.g., intensity‐modulated radiotherapy (IMRT)‐SBRT and volumetric‐modulated arc therapy (VMAT)‐SBRT), e) electromagnetic field fluctuations in SBRT that are specific to repeated beam holds (e.g., step‐and‐shoot IMRT and gating techniques) or nonconventional linac‐based technologies (e.g., continuous motion of the couch and gantry in tomotherapy with a shorter source‐to‐axis distance (SAD = 85 cm) instead of the usual 100 cm in conventional accelerators or the proximity/motion of CyberKnife linac relative to patients compared to conventional linac treatments), and f) extensive use of image‐guided radiotherapy (IGRT).^14^, ^15^

In the context of high dose per fraction, SBRT flattening filter‐free (FFF) beams have gained popularity.^19^, ^20^, ^21^ Flattening filter‐free beams have several unique features, including a lower out‐of‐field dose, a sharper penumbra, and less head scatter, which are potentially advantageous characteristics for patients with CIEDs.^14^ Additionally, FFF photon beams make a higher average dose rate possible, with an increase in the instantaneous dose rate for the dose pulses compared with conventional FF photon beams.^17^

Rodriguez et al.^22^ found that a transient effect on CIED can occur due to radiation‐induced photocurrents generated by a high‐dose rate. In newly published AAPM TG‐203,^17^ similar to the 2017 heart rhythm society (HRS) expert consensus statement on CIED,^23^ dose‐rate effect is considered as one of the risk factors for CIED function and fully discussed in this updated guideline. According to Hurkmans et al.,^24^ based on the theoretical failure mechanism, the effect of the pulse dose rate might be much more important than the average dose rate. However, the authors concluded that for conventional beams with flattening filter (FF) beams, the dose‐rate effect on CIEDs is not significant.^22^, ^24^ TG‐203 explicitly stated that the reported experience is based on conventional dose rate and there has not yet been a published research on possible effect of higher dose rate, higher dose per pulse, and higher dose per fraction in SBRT with FFF beams.

Several in vitro studies have been reported in the literature investigating the effect of RT with FF beam on CIEDs,^25^, ^26^, ^27^, ^28^, ^29^, ^30^ but there is a lack of robust data focusing on the effect of FFF beams using modern RT techniques on CIEDs.^31^ Additionally, given the increasing use of SBRT for both lung tumors and metastases, a study that specifically investigated CIED function during and after FFF beam irradiation would be of value.

In this study, a retrospective analysis of patients with CIEDs who underwent SBRT/SRS at Peter MacCallum Cancer Centre (abbreviated PeterMac) between 2014 and 2018 was performed. This was complemented through a phantom study to evaluate the effect of SBRT using FFF beams on CIED function.

## 2 Materials and methods

### 2.A Retrospective study

A retrospective review of all patients with CIEDs who underwent RT at the five campuses of PeterMac between 2014 and 2018 was performed. Then, data from CIED patients treated with SBRT/SRS, such as patient characteristics, type of CIEDs, irradiation sites, treatment plan specifications, and reports, were extracted from the MOSAIQ (Elekta AB, Stockholm, Sweden) database and Eclipse treatment planning system. Radiation oncologists were also asked to check their medical records and clinical notes to determine if any patients had a CIED malfunction and/or treatment complications during and/or after RT.

### 2.B Experimental study

#### 2.B.1 Device selection and programming

Recently explanted CIEDs from the Royal Melbourne Hospital Cardiology Department were analyzed to ascertain whether they had full functionality and a suitable battery life. Of these, 30 CIEDs (22 ICDs and 8 CRT‐Ds) were selected from two different manufacturers (Medtronic and St. Jude Medical [now Abbott]). None of the CIEDs were previously exposed to irradiation, and they could have been explanted for any reason except malfunction. All the CIEDs were programmed to a maximum and minimum frequency of 120 pulse per minute (ppm) and 60 ppm, respectively. The voltage and pulse width were also programmed to a fixed setting consistent with clinical outputs, and the sense threshold was set to the most sensitive level, approximately 0.2–0.3 mV. Shock therapy was deactivated.

#### 2.B.2 Interrogation before, during, and after radiation

To interrogate the devices, manufacturer‐specific programmers and a 4‐channel oscilloscope were used in this study. Before irradiation, parameters such as battery status and information including mode, longevity, and pacing pulse characteristics were obtained. Additionally, the sensing of each CIED was tested by injecting a 40 ms sine‐squared pulse or an ECG wave using a signal generator and an oscilloscope.^32^

**Fig. 1:**
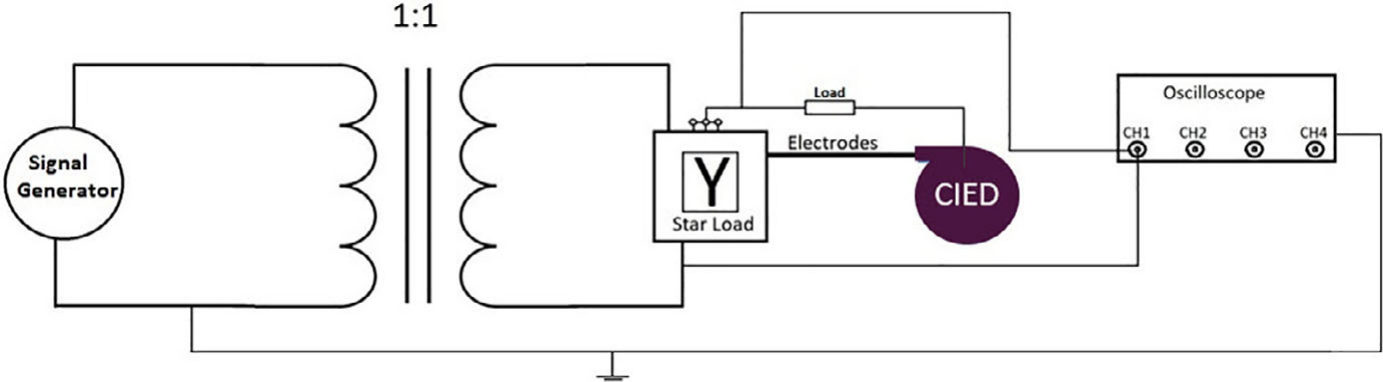
The isolation circuitry between the load and the oscilloscope was used to monitor the CIED during irradiation. A star load configuration with the electrodes, except the case, was tied to a common node through 250‐Ω resistors. The common node is tied back to the case via a 10‐Ω resistor.

The pacing output and sensitivity during irradiation were monitored by programmers and an oscilloscope to analyze the dose‐rate effect. An electrical load circuit was used to simulate myocardial resistance, and isolation circuitry between the load and oscilloscope was used, as recommended by the manufacturer’s principal R&D engineer (Fig. 1). The manufacturer‐specific programmers and oscilloscope were present in the bunker room to monitor the signal during irradiation. The oscilloscope was connected to a laptop placed in a control room. Additionally, the CIED marker channels were printed out using the programmer’s internal printer and were monitored using cameras in the radiotherapy bunker room.^33^ Inappropriate sensing during irradiation was determined by analysis of signals and marker channels using the programmers. The real signal (sin^2 or ECG) was also monitored within a couple of pacing cycles using the oscilloscope. Under‐sensing was reported in case of failing to see the signal and over‐sensing was reported in case of interpreting noise as a real signal. Finally, the CIEDs were interrogated after irradiation using the programmers and oscilloscope to check for any kind of malfunction (e.g., a loss of data or a reduction in the battery capacity) and any significant changes in the programmed CIED parameters (e.g., in the pulse voltage or in the pacing frequency).

#### 2.B.3 Experimental setup, planning, and irradiation

**Fig. 2:**
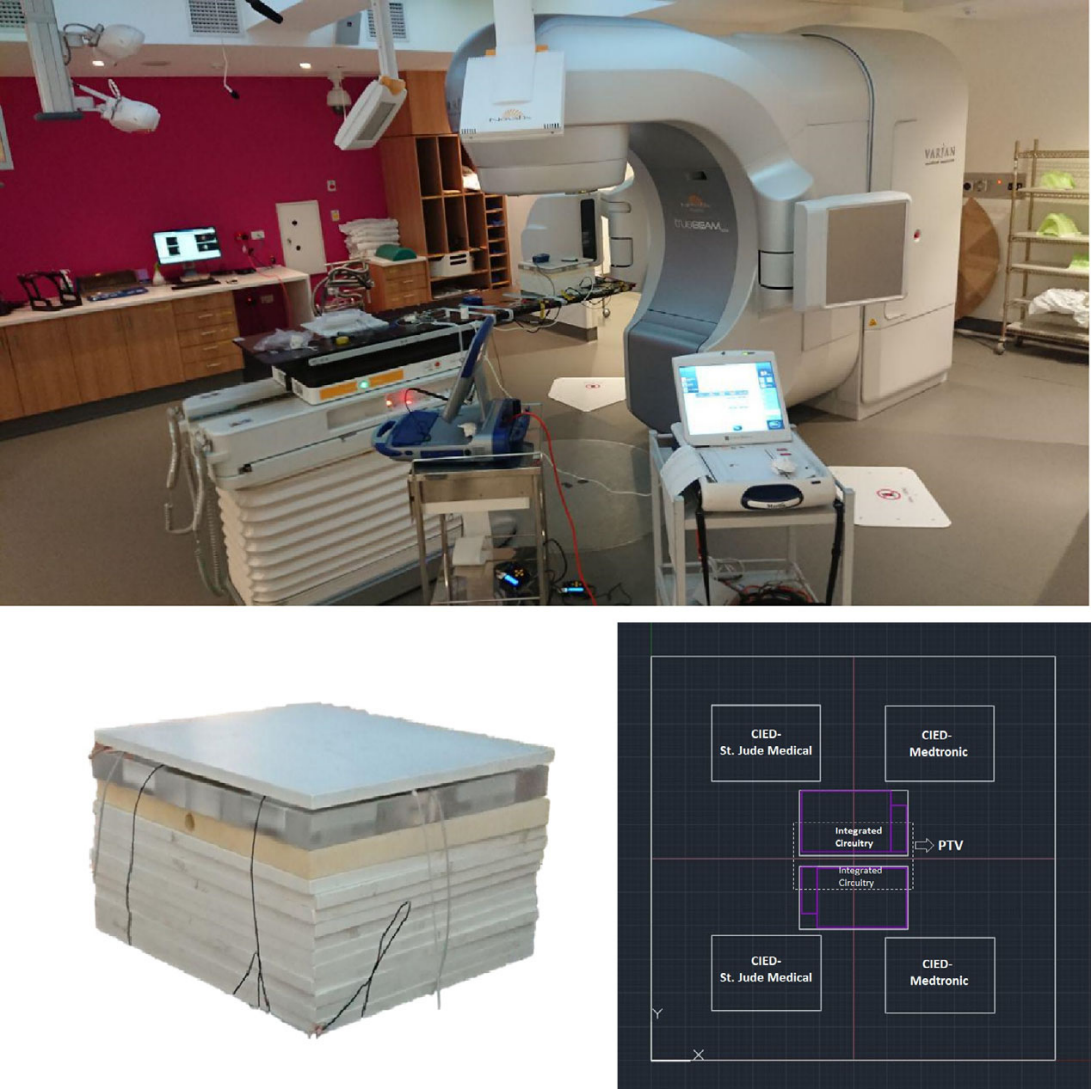
(Top) The experimental setup; (bottom left) The solid slab phantom; (bottom right) top view of the acrylic slab designed to position the CIEDs into six specific holes. Four holes were designed to be 3 cm away from the planning target volume (PTV), and two holes were designed to be placed partially inside the PTV.

Figure 2 depicts the experimental setup. The phantom included a 10‐cm water‐equivalent slab to provide full backscatter conditions. Then, an acrylic solid slab was designed to position the CIEDs. On top, a 1‐cm‐thick slab was used to simulate the patient’s tissue.^15^ The total size of the solid slab phantom was 30 cm × 30 cm × 15 cm.

Irradiation was performed using a Varian TrueBeam STx linac (Varian Medical Systems, Palo Alto, CA, USA) at PeterMac, Melbourne, Australia. First, a rep‐rate (=pulse repetition rate) test (RRT) including a range of beam pulse frequencies was conducted to determine when inappropriate sensing (either over‐sensing or inhibition) occurred. The maximum average dose rate and dose‐per‐pulse for TrueBeam in FFF mode are 14 Gy/min and 0.78 mGy/pulse for 6 MV and 24 Gy/min and 1.31 mGy/pulse for 10 MV beams, respectively.^34^ Our linear accelerator is calibrated to give 1 cGy per MU at depth Dmax for reference conditions of 100 cm SSD and 10 cm × 10 cm field size. The pulse repetition frequencies as a function of the average dose rate were measured in a previous study conducted by Biasi et al.^21^ The rep‐rate settings used in this study were tabulated in Table 1. In the RRT tests, the CIEDs (4 ICDs and 4 CRT‐Ds) were placed at a depth corresponding to the maximum dose and were directly irradiated with both 6‐MV FFF and 10‐MV FFF beams using a 10 cm × 10 cm field. The irradiation started with a low rep‐rate up to the highest value and delivered small doses to avoid exceeding the CIED’s total dose tolerance. Additionally, a few initial tests were performed with the device out of, but still near, the beam by moving the jaws to determine whether inappropriate sensing was due to electromagnetic interference (EMI).

**Table 1:** Pulse repetition frequency (Hz) for dose rate settings in TrueBeam in FFF mode.

Energy	Repetition rate (MU/min)	Pulse repetition frequency (Hz)
6 MV	1400	360
	1200	310
	1000	260
	800	206/207
	600	155
	400	206/207
10 MV	2400	360
	2000	300
	1600	240
	1200	180
	800	120
	400	120

MU/min, Monitor unit per minute; Hz, Hertz.

The second part of the irradiation process involved delivering four clinical treatment plans (CTPs) to the phantom. A solid slab was designed to place the CIEDs into six specific holes. The dimensions of the holes were based on the maximum dimensions of the selected CIEDs. Four holes were designed to be 3 cm away from the planning target volume (PTV), and two holes were designed to be placed partially inside the PTV. All the CIEDs were oriented and placed in such a way that their integrated circuitry was the most proximal part of the device to the PTV (Fig. 2). Experiments were repeated at least three times and mostly focused on monitoring during irradiation with the devices located outside the PTV.

Table 2 presents a summary of the rep‐rate tests (RRTs) conducted in this study. Table 3 illustrates the details of multifraction SBRT and single‐fraction SBRT treatment plans (SAFRON II)^35^ with FFF beams, which are frequently used at PeterMac for the treatment of lung tumors. The CT scan of the phantom was imported into the Varian Eclipse treatment planning system (version 15) with an Acuros XB algorithm (AXB1511). The CIEDs were contoured and overridden to an extended HU scale to perform the dose calculations.^36^

**Table 2:** Rep‐rate tests (RRTs).

	RRT #1	RRT #2
Site	–	–
Delivery technique	Open field irradiation at isocenter	Open field irradiation at isocenter
Energy & Beam mode	6 MV‐FFF	10 MV‐FFF
Dose/Fx	From 0.5 Gy up to 1.5 Gy	From 0.5 Gy up to 2 Gy
The maximum rep‐rate	600 MU/min 800 MU/min 1000 MU/min 1200 MU/min 1400 MU/min	400 MU/min 800 MU/min 1200 MU/min 1600 MU/min 2000 MU/min 2400 MU/min
Position of integrated circuits to PTV	▪ Inside	▪ Inside
CIED #	▪ 4 CIEDs (Inside) **CIED #1:** ICD (Medtronic) **CIED #2:** ICD (St. Jude Medical) **CIED #3:** CRT‐D (St. Jude Medical) **CIED #4:** CRT‐D (Medtronic)	▪ 4 CIEDs (Inside) **CIED #5:** ICD (St. Jude Medical) **CIED #6:** ICD (Medtronic) **CIED #7:** CRT‐D (St. Jude Medical) **CIED #8:** CRT‐D (Medtronic)

CIED, Cardiac implantable electronic device; CRT‐D, Cardiac resynchronization therapy defibrillator; FFF beam, Flattening‑filter‑free beam; ICD, Implantable cardioverter defibrillator; MU/min, Monitor unit per minute; PTV, Planning target volume; RRT, Repetition rate test.

**Table 3:** Clinical treatment plans (CTPs).

	CTP #1	CTP #2	CTP #3	CTP #4
Site	Lung and Chest	Lung and Chest	Lung and Chest	Lung and Chest
Delivery technique	VMAT‐SBRT	VMAT‐SBRT	VMAT‐ SBRT	3DCRT‐ SBRT
Energy & Beam mode	10 MV‐FFF	6 MV‐FFF	10 MV‐FFF	6 MV‐FFF
Dose/Fx	28 Gy/1 Fx	28 Gy/1 Fx	48 Gy/4 Fx	48 Gy/4 Fx
The maximum rep‐rate	2400 MU/min	1400 MU/min	2400 MU/min	1400 MU/min
Position of integrated circuits to PTV	Partially inside Outside: 3 cm away	Partially inside Outside: 3 cm away	Partially inside Outside: 3 cm away	Partially inside Outside: 3 cm away
CIED #	2 CIEDs (Partially inside) **CIED #9:** ICD (Medtronic) **CIED #10:** ICD **(**St. Jude Medical) 4 CIEDs (Outside) **CIED #11:** CRT‐D (Medtronic) **CIED #12:** ICD (St. Jude Medical) **CIED #13:** ICD (Medtronic **CIED #14:** ICD (St. Jude Medical)	2 CIEDs (Partially inside) **CIED #15:** ICD (Medtronic) **CIED #16:** ICD (St. Jude Medical) 3 CIEDs (Outside) **CIED #17:** ICD (Medtronic) **CIED #18:** ICD (St. Jude Medical) **CIED #19:** CRT‐D (Medtronic)	2 CIEDs (Partially inside) **CIED #20:** ICD (St. Jude Medical) **CIED #21:** ICD (Medtronic) 4 CIEDs (Outside) **CIED #22:** ICD (St. Jude Medical) **CIED #23:** ICD (Medtronic) **CIED #24:** CRT‐D (St. Jude Medical) **CIED #25:** ICD (Medtronic)	2 CIEDs (Partially inside) **CIED #26:** ICD (St. Jude Medical) **CIED #27:** ICD Medtronic) 3 CIEDs (Outside) **CIED #28:** ICD (St. Jude Medical) **CIED #29**: ICD (Medtronic) **CIED #30:** CRT‐D (St. Jude Medical)

CTP Clinical treatment plan; VMAT‐SBRT, Volumetric‐modulated arc therapy‐Stereotactic body radiotherapy; 3DCRT‐SBRT, Three‐dimensional conformal radiation therapy‐ Stereotactic body radiotherapy; PTV, Planning target volume; MU/min, Monitor unit per minute; CIED, Cardiac implantable electronic device; ICD, Implantable cardioverter defibrillator; CRT‐D, Cardiac resynchronization therapy defibrillator.

## 3 Results

### 3.A Retrospective study

**Fig. 3:**
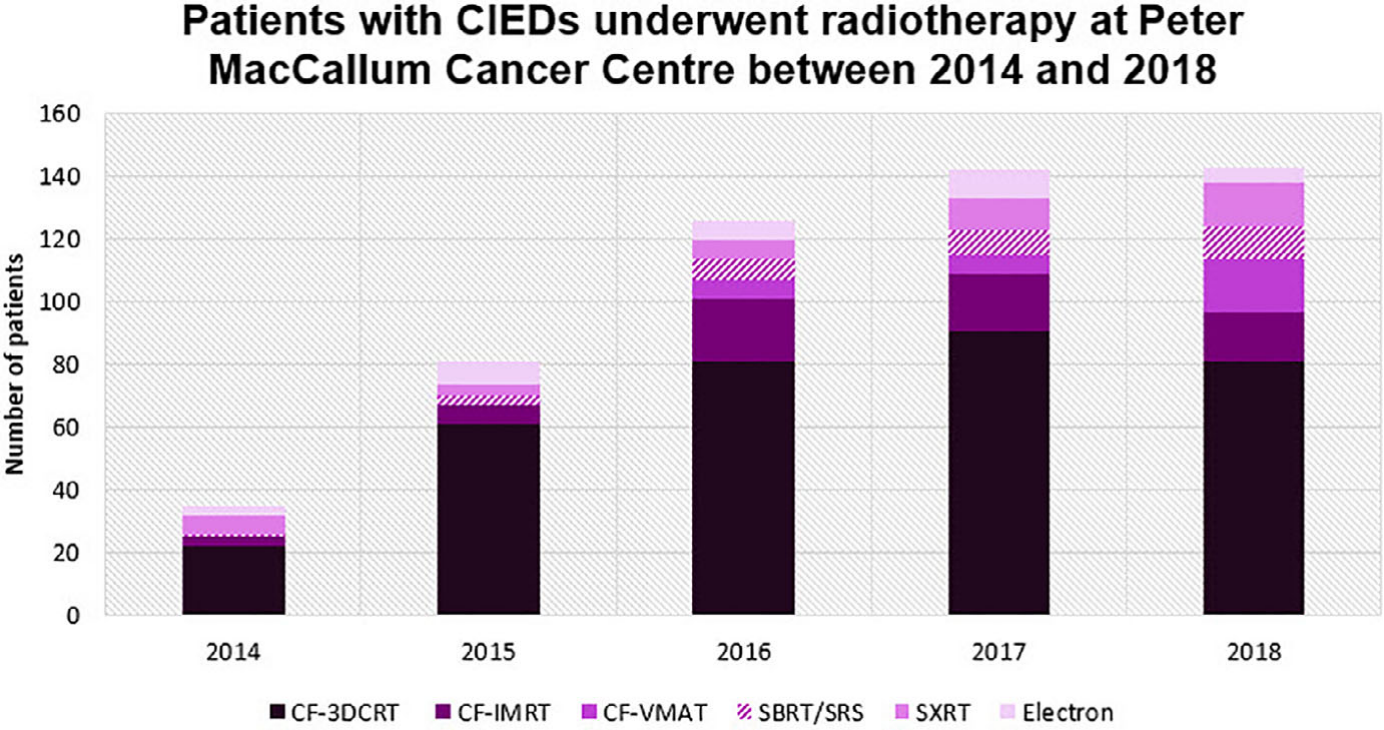
A retrospective analysis of patients with CIEDs undergoing radiotherapy at PeterMac between 2014 and 2018; CF‐3DCRT: Conventionally fractionated three‐dimensional conformal radiotherapy, CF‐IMRT: Conventionally fractionated intensity modulated radiotherapy, CF‐VMAT: Conventionally fractionated volumetric modulated arc therapy, SBRT/SRS: Stereotactic body radiotherapy/Stereotactic radiosurgery, SXRT: Superficial x ray radiation therapy.

In total, 523 patients with CIEDs who received at least one course of RT at the five campus of PeterMac between 2014 and 2018 were included in the study. As shown in Fig. 3, although the majority of CIED patients were treated with three‐dimensional conformal radiotherapy (3DCRT) during the study period, there was a continuous increase in the number of patients who had CIEDs and underwent SBRT/SRS.

Of the 28 patients received SBRT/SRS, there were 23 pacemaker cases, 4 ICD cases, 1 CRT‐D case, and none were pacing‐dependent. Thirteen cases were treated with FFF beams at rep‐rate of 2400 MU/min (11 cases), as well as 1400 MU/min (2 cases), and 15 cases were treated with FF beams at rep‐rate of 600 MU/min. Three ICD cases were treated with conventional 6‐MV FF beams; treatment sites were right lung and kidney. The closest distance between the radiation field and CIEDs was 8 cm (right lung), and more than 10 cm (kidney). The last ICD case was a right‐lung tumor patient who received SBRT‐FFF with 10 MV‐FFF and a maximum rep‐rate of 2400 MU/min. The ICD (Boston Scientific‐VENTAK PRIZM) was implanted in the left side of the chest and ICD to PTV distance was more than 10 cm. A total prescription dose of 48 Gy [12 Gy*4 fractions] was delivered to the patient using VMAT with two coplanar arcs and max CIED dose was estimated to receive less than 20 cGy. The only CRT‐D case was a patient with right lung cancer, and received VMAT‐SBRT with 10 MV‐FFF and a maximum rep‐rate of 2400 MU/min. The CRT‐D (Viva Quad XT) was more than 10 cm away from the treatment field and maximum estimated dose to CRT‐D was negligible (less than 10 cGy).

**Fig. 4:**
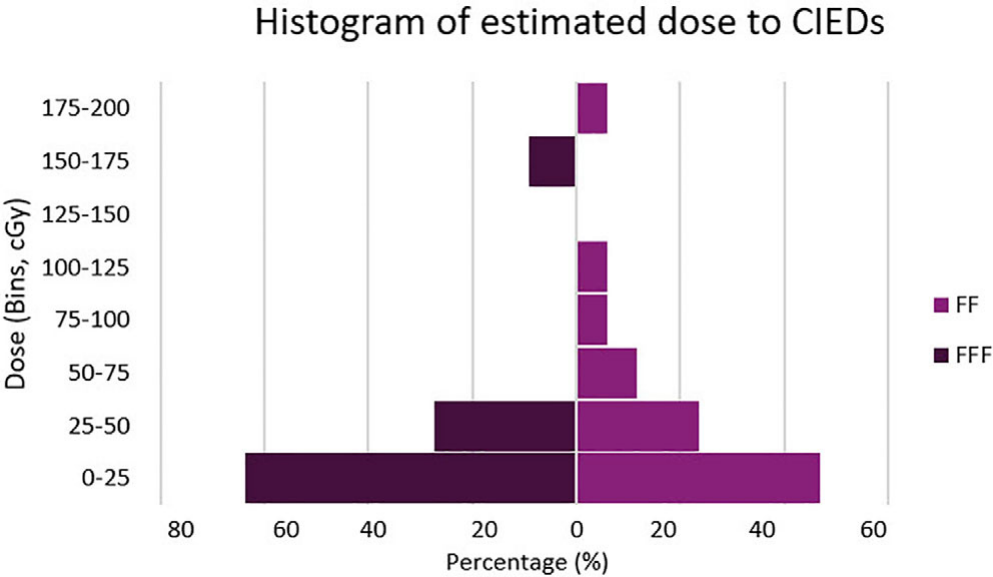
Histograms show estimated dose to CIEDs from FF and FFF beams. The median (range) calculated dose to CIEDs was 0.11 (0–1.5) Gy for FFF beams and was 0.22 (0.01–1.86) for FF beams.

The characteristics and treatment plans of all CIED patients who were treated with SBRT/SRS are summarized in Table 4. All of the devices were positioned outside the treatment field at a distance greater than 5 cm with the exception of one case where the distance was 4 cm. The median (range) calculated dose the CIEDs were exposed to was 0.2 (0–1.86) Gy (Fig. 4). The functionality of the CIEDs during and after RT was investigated, and no failures were reported due to SBRT/SRS.

**Table 4:** Summary of the clinical treatment plans and their related features.

Characteristics	Study population	SBRT/SRS with FFF	SBRT/SRS with FF
No.	28	13	15
Patient age, median (range), y	80 (57–93)	–	–
Type of CIED, No.			
PM	23	11	12
ICD	4	1	3
CRT‐D	1	1	0
Site of irradiation, No.			
Lung and chest	17	11	5
Brain	2	0	2
Liver	2	1	1
Kidney	5	0	5
Spine	1	0	1
Pelvis	1	1	1
Treatment plan specification photon energy			
6 MV	17	2	15
10 MV	11	11	0
Number of fraction, median (range)	3 (1–5)	–	–
Dose/fraction, median (range), Gy	29 (28–54)		
Rep‐rate (MU/Min)			
2400 (MU/Min)	11	11	0
1400 (MU/Min)	0	2	0
600 (MU/Min)	15	0	15
Distance between CIED and closest treatment field edge			
less than 5 cm	1	1	0
5–10 cm	4	0	4
More than 10 cm	23	10	13
SBRT delivery technique			
VMAT	11	4	7
3DCRT	17	4	13

CIED, Cardiac implantable electronic device; PM, Pacemaker; ICD, Implantable cardioverter defibrillator; CRT‐D, Cardiac resynchronization therapy; SBRT, Stereotactic body radiotherapy; SRS, Stereotactic radiosurgery; FFF beam, Flattening‑filter‑free beam; FF beams flattened beams.

### 3.B Experimental study

#### 3.B.1 Rep‐Rate Tests (RRTs)

**Fig. 5:**
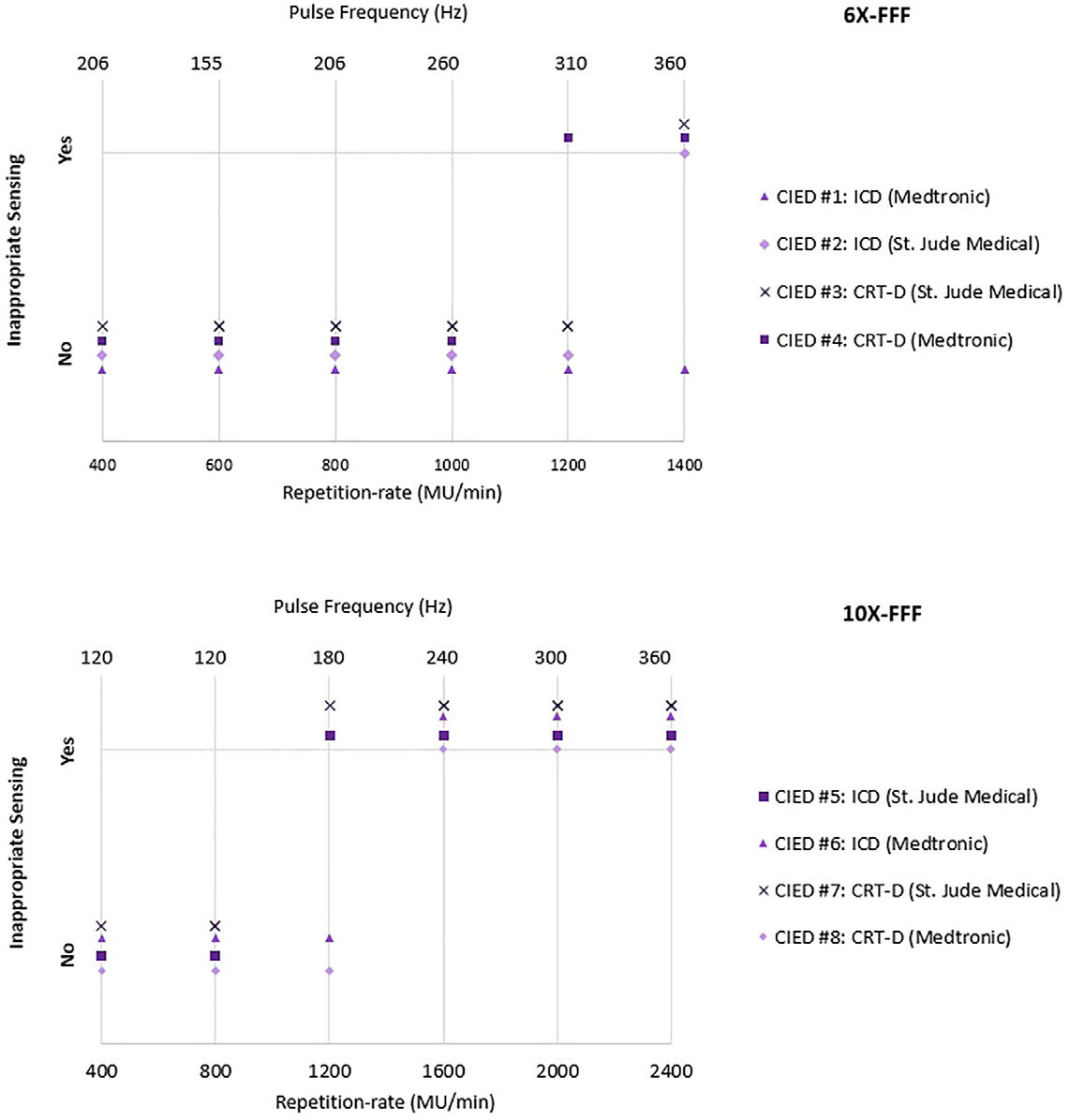
Results of the rep‐rate test for 6‐MV FFF (up) and 10‐MV FFF (down) to determine when inappropriate sensing (either over‐sensing or inhibition) occurs.

A rep‐rate of less than 1200 MU/min for both the 6‐MV FFF and 10‐MV FFF beams did not affect the sensing function of the selected CIEDs. Inappropriate sensing started to detect somewhere between 1200 and 1600 MU/min. (Fig. 5).

#### 3.B.2 Clinical treatment plans (CTPs)

The CIED dysfunctions observed during and after delivering lung SBRT from the first set of irradiation to the phantom are summarized in Table 5. No errors were observed in the CIEDs that were irradiated using CTP #2 and CTP #4 with 6‐MV FFF photon beams. During the delivery of CTP #1 and CTP #3 with 10‐MV FFF photon beams, inappropriate sensing occurred in CIED #10, CIED #13, and CIED #20. Note that over‐sensing was minor and did not meet criteria for a shock. Interrogation after irradiation showed changing in programmed parameters such as pacing mode, ventricular pacing threshold, pacing rate, and pulse amplitude. Inadequate shock therapy (in spite of deactivation) and battery voltage changes/longevity were not detected by the programmers. Additionally, no permanent damage to the CIEDs was reported.

**Table 5:** Types of CIED errors.

CIED #	CTP #	Position of electronic circuit of CIED to PTV	Inappropriate sensing during irradiation	Interrogation after irradiation	Total calculated dose to	
					the part of device inside the field including integrated circuits (Gy)	the part of device outside the field (Gy)
9	1	Partially inside	No	Reprogramming of pacing rate	Dmax: 26.1 Dmean: 16.2	Dmax: 17.8 Dmean: 5.6
10	1	Partially inside	Yes	Reprogramming of pacing rate	Dmax: 28 Dmean: 16.9	Dmax: 18.7 Dmean: 2.8
20	3	Partially inside	Yes	A decrease in pulse amplitude after delivering the last fraction	Dmax: 46.4 Dmean: 28.8	Dmax: 30.7 Dmean: 9.6
21	3	Partially inside	No	Reprogramming of ventricular pacing threshold	Dmax: 42.7 Dmean: 28.4	Dmax: 29.2 Dmean: 4.8
					Total calculated dose to the CIED (Gy)	
13	1	Outside	Yes	Change in pacing mode	Dmax: 0.89 Gy Dmean: 0.66 Gy	
24	3	Outside	No	Change in pacing mode	Dmax: 0.96 Gy Dmean: 0.85 Gy	

CTP, Clinical treatment plan; CIED, Cardiac implantable electronic device; PTV, Planning target volume; Dmax, Maximum dose; Dmean Mean dose.

Similar results were obtained in repeated experiments with CIEDs positioned outside the beam and no inappropriate sensing occurred during delivery of CTP #2, CTP #3, and CTP #4.

**Fig. 6:**
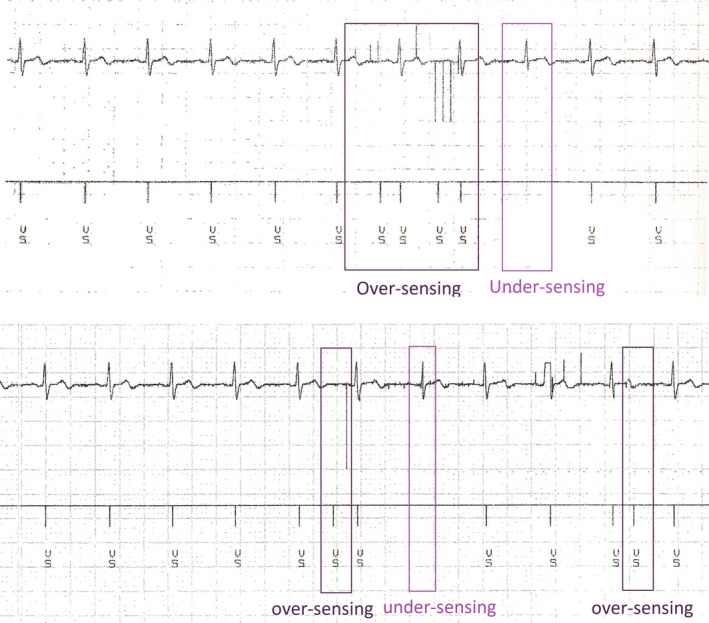
The CIED electrograms demonstrates transient inappropriate sensing during the delivery of CTP #1.

To confirm the results obtained from CTP #1, the experiment was repeated with 13 remaining CIEDs (six Medtronic CIEDs and seven St. Jude Medical CIEDs) which did not exceed CIED’s total dose tolerance. They were setup with the conditions that CEID #13 failed and were irradiated with the same plan CTP #1. In total, by also including CIED #13, three inappropriate sensing detected during irradiation of CTP #1 (Fig. 6).

It should be noted that despite CEID #13 and CEID #24 showed errors, we centered the analysis on the position of CIED #13 where the error detected during the irradiation. However, it is recommended to check the CEIDs after the irradiation.

## 4 Discussion

With the widespread implementation of advanced RT technologies and techniques, including SBRT treatments with FFF beams, the need to consider the different challenges of these techniques when managing patients with CIEDs has arisen.^14^, ^37^

TG 203 and other guidelines highly recommended that direct irradiation of the device should be avoided. In the rep‐rate tests conducted in this study, CIED were located directly within the radiation field, which may not be the clinical situation. This test, however, was designed to better understand the frequency and severity of failure caused by irradiation from high‐dose rate of the FFF beams. Different factors, such as the CIED cumulative dose,^27^ the radiated EMI problem, and the CIED programming and model, should be taken into account to accurately evaluate dose‐rate effects. According to our experiments, failure due to dose‐rate effects were detected for both models from two different manufacturers, with inappropriate sensing start to detect somewhere between 1200 and 1600 MU/min. This dose rate, however, should not be considered as a general threshold below which no inappropriate sensing occurs in all ICD and CRT‐D models and setup conditions.

Inappropriate sensing includes both over‐ and under‐sensing. In our test, it was observed that over‐sensing was more frequent for rep‐rate ranging between 1200 MU/min and 2000 MU/min, and under‐sensing or loss of sensing was more frequent for rep‐rates greater than 2000 MU/min. Although any type of sensing abnormalities should be taken into consideration, over‐sensing can become a more concerning issue for pacing‐dependent patients.^38^

Several in vitro studies concerning CIED irradiation using FF beams have been performed.^25^, ^26^, ^27^, ^28^, ^29^, ^30^ Mouton et al.^27^ conducted an in vitro study using a Saturne 3 linac (CGR MeV‐General Electric). The irradiation was performed at 18 MV and with different dose rates up to 8 Gy/min. They recommended a maximum dose rate of 0.2 Gy/min, rejecting the direct irradiation of the pacemaker, at a standard dose rate for tumor treatment (2 Gy/min). Hurkmans et al.^24^ concluded that for conventional FF beams, the dose‐rate effect (e.g., ranging from 1 Gy/min to 6 Gy/min at a depth corresponding to the maximum dose at a reference distance) on CIEDs is not significant because the dose rate at the CIED location is lower than the recommended maximum acceptable value.^27^ Hurkmans and colleagues^24^ further added that for FFF beams, the dose rate would be lower than 1 Gy/min, provided that the CIED is located outside of the treatment field. Thus, the dose‐rate effect is rare. Gauter‐Fleckenstein et al.^31^ published the first in vitro study of ICDs in which the authors used FFF. Irradiation was conducted with a cumulative dose of up to 150 Gy in the isocenter, [10 Gy*3 fractions] in five sets, with 6‐, 10‐ and, 18‐MV beams. Their results showed that during 6‐MV FFF‐VMAT, the risk of CIED malfunctions is very low even when cardiac devices are located in the close vicinity of the PTV. CIED anomalies during 10‐MV FFF‐VMAT were, however, observed.

In our clinical review, all of the devices were positioned at least 4 cm, but typically > 5 cm, from the PTV. Despite high energy and high‐dose rate used, no effects were found. The second part of our irradiation experiment attempted to imitate a real‐world SBRT scenario, including clinical fractionation, dose rates, and delivery techniques. In general, our results suggested that in SBRT treatments with FFF beams the effect of a high‐dose rate is infrequent even when the CIED is in the vicinity of the PTV. Among the result, the most interesting situation was transient inappropriate sensing detected in CEID #13. In this scenario, the CEID was situated outside the PTV, irradiated with 10 MV‐FFF, and the estimated dose to CIED dose was less than 2 Gy. Of the 13 extra CEIDs that were setup with the conditions that CEID #13 failed and were irradiated with the same plan CTP #1, two transient inappropriate sensing were detected (13 CIEDs/2 inappropriate sensing). However, our data do not allow definite conclusions on whether the detected issues are as a result of high‐dose rate, high energy, or other causes.

An analysis for maximum doses received by CIEDs (located outside the beam) for all CTPs confirmed that they are all lower than the historical threshold dose of 2 Gy. As can be seen from Table 5, CIED #13 experienced anomalies with a maximum dose of 0.89 Gy. No dysfunction was observed in CIED #11 and CIED #14 that were irradiated using CTP #1 and received maximum doses of 0.93 Gy and 1.02 Gy, respectively. Therefore, dose limit of 2Gy, and in general risk stratification based on a dose threshold, cannot be a sufficient estimator, as observed in CTP #1 with high energy.

As detailed by AAPM TG‐203,^17^ and other studies,^11^, ^24^, ^39^ there is no safe dose threshold below which no CIED damage occurs. However, because increasing the cumulative dose increases the risk of failure, the consensus is that the cumulative dose received by CIEDs should be kept as low as possible.^15^, ^24^

In the new AAPM guideline,^17^ CIED patients are risk‐categorized not only on the basis of the CIED cumulative dose and pacing dependency^15^, ^24^ but also on the basis of neutrons present; the risk categories are low (the CIED dose is less than 2 Gy and the patient is not pacing‐dependent), medium (CIED dose is between 2 Gy and 5 Gy and the pacing‐dependent patients receive a CIED dose less than 2 Gy), and high (patients who receive a CIED dose above 5 Gy or patients treated with high‐ energy photons (energies >10 MV) or protons with neutrons are considered to be present).^17^

In general, SBRT/SRS is applied to small treatment volumes; near the target volume, lower doses are produced by flattening filter‐free‐beam in comparison to that produced by the FF‐beam.^18^, ^19^ In this study, the CIED errors mostly occurred when the devices were positioned partially inside the beam and electronic circuit of CIED received at least 80% of the prescribed dose. However, during and after delivering lung SBRT from the first set of irradiations, two devices (CIED #13 and CIED #24) that were positioned outside the beam and were irradiated with 10‐MV FFF‐VMAT showed three errors at doses lower than 2 Gy. Therefore, according to the TG‐203, direct irradiation (or even partially irradiation) should be avoided and attention should be paid for scattered radiation outside the field.

As discussed in the TG‐203, and a recent review paper^14^ focusing on SBRT/SRS and CIEDs, it is not clear that one needs to consider dose/min, dose/s, dose/pulse, or dose/fraction. In this study, CIEDs were exposed to a high dose per fraction. Although this exposure can result in a higher CIED dose per fraction,^14^, ^24^ the increased dose per fraction by itself is not a serious concern, as long as the maximum accumulated dose to the devices is lower than the recommended value. However, the use of FFF beams with energies greater than 6 MV and high dose per fraction can increase the risk of CIED malfunctions, including inappropriate sensing and reprogramming, in close vicinity of the PTV.

As with other studies, this research is also subject to limitations. The first limitation of this study was that the tested CIEDs were from only two different manufacturers, which limited the results and the analysis. Additionally, it was suggested that the device’s projected battery life and current status were not appropriate surrogates in this test, and instead, actual measurements are required.

## 5 Conclusion

A retrospective analysis of patients with CIEDs who underwent radiotherapy at the Peter MacCallum Cancer Centre demonstrated an increase in the number of these patients who received conventional linac‐based SBRT and SRS. Thus, some of the potential patient risk factors, such as a high dose per fraction, a high‐dose rate using FFF beams with two different energies, and delivery with an intensity‐modulated technique, were investigated in this study.

The study showed that caution is needed to manage CIED patients undergoing SBRT with FFF beams, as it is recommended by AAPM TG‐203.^17^ Correspondingly, it was found that with FFF beams, although there is small risk from dose‐rate effects, delivering high dose of radiation with beam energy greater than 6 MV and high‐dose rate to a CIED positioned in the close vicinity of target may present issues.

The results of this study and a recent review study^14^ were used to update some of the policies applied to manage CIED patients undergoing SBRT/SRS at PeterMac. According to this update, CIEDs should not be even placed partially inside the SBRT treatment field. Also, for patients who are pacing‐dependent and have CIEDs positioned in close vicinity of the PTV, the use of FFF with 10 MV beams should be restricted.

## Conflict of interest

All the authors declare no conflict of interest.
